# Are there differences among operators in false-negative rates of endosonography with needle aspiration for mediastinal nodal staging of non-small cell lung cancer?

**DOI:** 10.1186/s12890-018-0774-6

**Published:** 2019-01-14

**Authors:** Sukyeon Kim, Beomsu Shin, Hyun Lee, Jick Hwan Ha, Kyungjong Lee, Sang-Won Um, Hojoong Kim, Byeong-Ho Jeong

**Affiliations:** 1 Division of Pulmonary Medicine, Department of Internal medicine, Hangang Sacred Heart Hospital, Hallym University School of Medicine, Seoul, Republic of Korea; 2 Department of Internal Medicine, Yonsei University Wonju College of Medicine, Wonju, Republic of Korea; 3 Division of Pulmonary Medicine and Allergy, Department of Internal Medicine, Hanyang University College of Medicine, Seoul, Republic of Korea; 4 Division of Pulmonology, Critical Care and Sleep Medicine, Department of Internal Medicine, College of Medicine, The Catholic University of Korea, Seoul, Republic of Korea; 5 Division of Pulmonary and Critical Care Medicine, Department of Medicine, Samsung Medical Center, Sungkyunkwan University School of Medicine, Irwon-ro 81, Gangnam-gu, Seoul, 06351 Republic of Korea

**Keywords:** Endosonography with needle aspiration (EBUS/EUS-NA), Non-small cell lung cancer, False negative rate, Operator factor

## Abstract

**Background:**

Endosonography with needle aspiration (EBUS/EUS-NA) is recommended as the first choice for mediastinal nodal assessment in non-small cell lung cancer (NSCLC). It is important to maintain adequate negative predictive value of the procedure to avoid unnecessary additional surgical staging, but there are few studies on the influence of operator-related factors including competency on false negative results. This study aims to compare the false negative rate of individual operators and whether it changes according to accumulation of experience.

**Methods:**

This is a retrospective study of NSCLC patients who were N0/N1 by EBUS/EUS-NA and confirmed by pathologic staging upon mediastinal lymph node dissection (*n* = 705). Patients were divided into a false negative group (finally confirmed as pN2/N3) and a true negative group (pN0/N1). False negative rates of six operators and whether these changed according to accumulated experience were analyzed.

**Results:**

There were 111 (15.7%) false negative cases. False negative rates among six operators ranged from 8.3 to 21.4%; however, there were no statistical differences before and after adjustment for patient characteristics and procedure-related factors (*P* = 0.346 and *P* = 0.494, respectively). In addition, false negative rates did not change as each operator accumulated experience (*P* for trend = 0.632).

**Conclusions:**

Our data suggest that there would be no difference in false negative rates regardless of which operator performs the procedure assuming that the operators have completed a certain period of observation and have performed procedures under the guidance of an expert.

**Electronic supplementary material:**

The online version of this article (10.1186/s12890-018-0774-6) contains supplementary material, which is available to authorized users.

## Introduction

Clinical tumor-node-metastasis (TNM)-staging in non-small cell lung cancer (NSCLC) is a pivotal factor for deciding the treatment plan and assessing prognosis [1, 2]. Although computed tomography (CT) and positron emission tomography (PET) are recommended for evaluation of TNM staging, invasive mediastinal staging techniques are still the gold standard approach for confirming N-staging because imaging techniques have low sensitivity and specificity to predict metastasis to mediastinal and hilar lymph nodes (LNs) [1]. Recently, endosonography (endobronchial ultrasound [EBUS] and esophageal ultrasound [EUS]) with needle aspiration (EBUS/EUS-NA) was recommended as the first choice for mediastinal nodal assessment over surgical staging because of its cost effectiveness, minimal invasiveness, and high sensitivity for evaluation of N-stage in patients with NSCLC [1, 3, 4].

However, this recommendation is based on the operator having appropriate experience and skill level. The experience of the operator performing the needle biopsy is known to have a significant impact on the diagnostic yield [5–7]. Moreover, additional surgical N-staging such as mediastinoscopy is recommended if the results of EBUS/EUS-NA are negative but there are suspicions of mediastinal nodal metastasis because the sensitivity of EBUS/EUS-NA is not high enough to completely rule out nodal metastasis [1, 4, 8]. Therefore, it is important to manage the test quality of EBUS/EUS-NA to achieve appropriate diagnostic yield, reduce false negative results, and avoid unnecessary surgical N-staging.

There are many published studies about patient-related and tumor-related predictors for false negative results of EBUS/EUS-NA [9, 10], but few reports about operator-related factors. Although many studies have evaluated how fast individual trainees adjust to the procedure as assessed by various evaluation tools [7, 11, 12], there is a lack of studies analyzing the false negative rates of EBUS/EUS-NA depending on operator and using surgical staging as the reference value. In this study, we aim to evaluate the false negative results of EBUS/EUS-NA to N2 or N3 LNs depending on operator and accumulated experience.

## Methods

### Study population

This study was retrospectively conducted by reviewing the medical records of patients who had histologically confirmed primary NSCLC at Samsung Medical Center, a tertiary referral hospital in South Korea, between March 2013 and November 2016. Based on CT and PET images, LN involvement was determined according to the nodal definition of the International Association for the Study of Lung Cancer (IASLC) [2]. All patients who were suspected to have clinical N3, N2, and N1 nodes which were defined as short-axis of LNs > 1 cm in CT or maximum standardized uptake value of LNs > 2.5 in PET [13], or whose tumor was larger than 3 cm or centrally located underwent preoperative tissue confirmation with EBUS/EUS-NA for nodal staging according to recent guidelines [1, 4].

Over the study period, patients with NSCLC who had negative results of EBUS/EUS-NA for N2 and N3 were enrolled. Among these patients, those who did not undergo definitive surgery with mediastinal dissection or surgical staging were excluded. We investigated baseline characteristics of the patients including age, sex, and location and histologic type of primary tumor, contents of procedure such as assessed LN station, duration of procedure, combination of EUS, nodal size, and number of aspiration and obtained core tissue per node, and final results of surgical staging. This study was approved by the Institutional Review Board of Samsung Medical Center (IRB No.2017–02–075-011). Informed consent was waived because of the retrospective nature of the study.

### EBUS/EUS-NA

During the study period, there were six operators with different experience levels of EBUS/EUS-NA (Additional file 1: Figure S1). We designated the operators as A to F according to the number of performed procedures. All operators had at least 2 years of bronchoscopy experience and started performing the EBUS/EUS-NA procedure independently without a supervisor after at least 200 cases of observation and assistance over a period of 6 months. Operator A participated for the entire period of this study and performed 1228 cases of EBUS/EUS-NA. Of these cases, 290 cases with negative results of EBUS/EUS-NA for nodal staging of NSCLC patients were analyzed. Operators B and D already had many experiences with the EBUS/EUS-NA procedure at the start of this study, and performed 1072 and 279 cases of EBUS/EUS-NA during the study period, respectively. Of these cases, 219 (operator B) and 67 (operator D) were analyzed for the purpose of this study, respectively. Operators C, E, and F started performing the EBUS/EUS-NA procedure in the middle of the study, and they performed 368, 143, and 84 cases of EBUS/EUS-NA during the study period, respectively. Of these cases, 84, 33, and 12 cases were analyzed for operator C, E, and F, respectively.

EBUS/EUS-NA was performed with a convex probe-EBUS bronchoscope (BF-UC260F-OL8; Olympus, Tokyo, Japan) and a 22-gauge needle (NA-201SX-4022; Olympus) [8, 14]. The mediastinal and hilar LNs were systematically and completely assessed in the standard sequence from N3 to N2 to N1 [15]. We have performed EBUS through the airway, and if necessary, EUS through esophagus using EBUS scope could be added for the specific LN stations. However, LNs in N1 were completely evaluated only if a patient had a difference in treatment plan depending on N1 metastasis. We performed EBUS/EUS-NA under moderate sedation with intravenous midazolam and fentanyl. Rapid on-site cytolopathological examination (ROSE) was not available during this study period.

To assess the accuracy of EBUS/EUS-NA, final surgical pathology from mediastinal LN dissection (MLND) was considered the gold standard. If final surgical pathology was N0 or N1, we assessed the result of EBUS/EUS-NA as true negative. If final surgical pathology was N2 or N3, we assessed the result of EBUS/EUS-NA as false negative. LNs with false negative results based on EBUS/EUS-NA were further classified as attempted LNs, inaccessible LNs, and unattempted accessible LNs. Attempted LNs were defined as LNs that were actually obtained during EBUS/EUS-NA. LNs in stations 3A, 5, and 6, which are not easily reached with the needle in EBUS/EUS-NA, were defined as inaccessible [16]. Unattempted accessible LN was defined as an LN that was easily accessible during the procedure but was not examined according to the operator’s clinical decision.

### Statistical analysis

Data are presented as numbers and percentages for categorical variables and as means and standard deviations for continuous variables. Categorical variables were compared using Pearson’s chi-square test or Fisher’s exact test. Continuous variables were compared using Student’s T-test or ANOVA test and post-hoc analysis.

Patient characteristics, contents of the procedure, and false negative results were separately assessed according to patient and LN. All results were re-analyzed after excluding patients with false negative results of EBUS/EUS-NA on the inaccessible LNs when assessed by patient and after adding unattempted accessible LNs when assessed by LN.

Multivariable logistic regression analysis was used to adjust for potential confounding factors in the association between false negative results of EBUS/EUS-NA and operator factors, using operator A as the reference category. Three models were constructed: Model 1 was adjusted for patient characteristics such as age, sex, location and histologic pattern of tumor; Model 2 was adjusted for contents of procedure such as duration of procedure and combination of EUS, and number of evaluated lesions for analyzing by patient or nodal size (short axis), number of aspiration per node, and number of obtained core tissue per node for analyzing by LN; Model 3 was adjusted for both patient characteristics and contents of procedure. We also assessed whether false negative rates of EBUS/EUS-NA decreased according to increased operator experience by the trend test (Mantel–Haenszel test). All tests were two-sided, and *P* values < 0.05 were considered statistically significant. All data were analyzed using PASW software (IBM SPSS Statistics ver. 22, Chicago, IL, USA).

## Results

### Study population

**Fig. 1:**
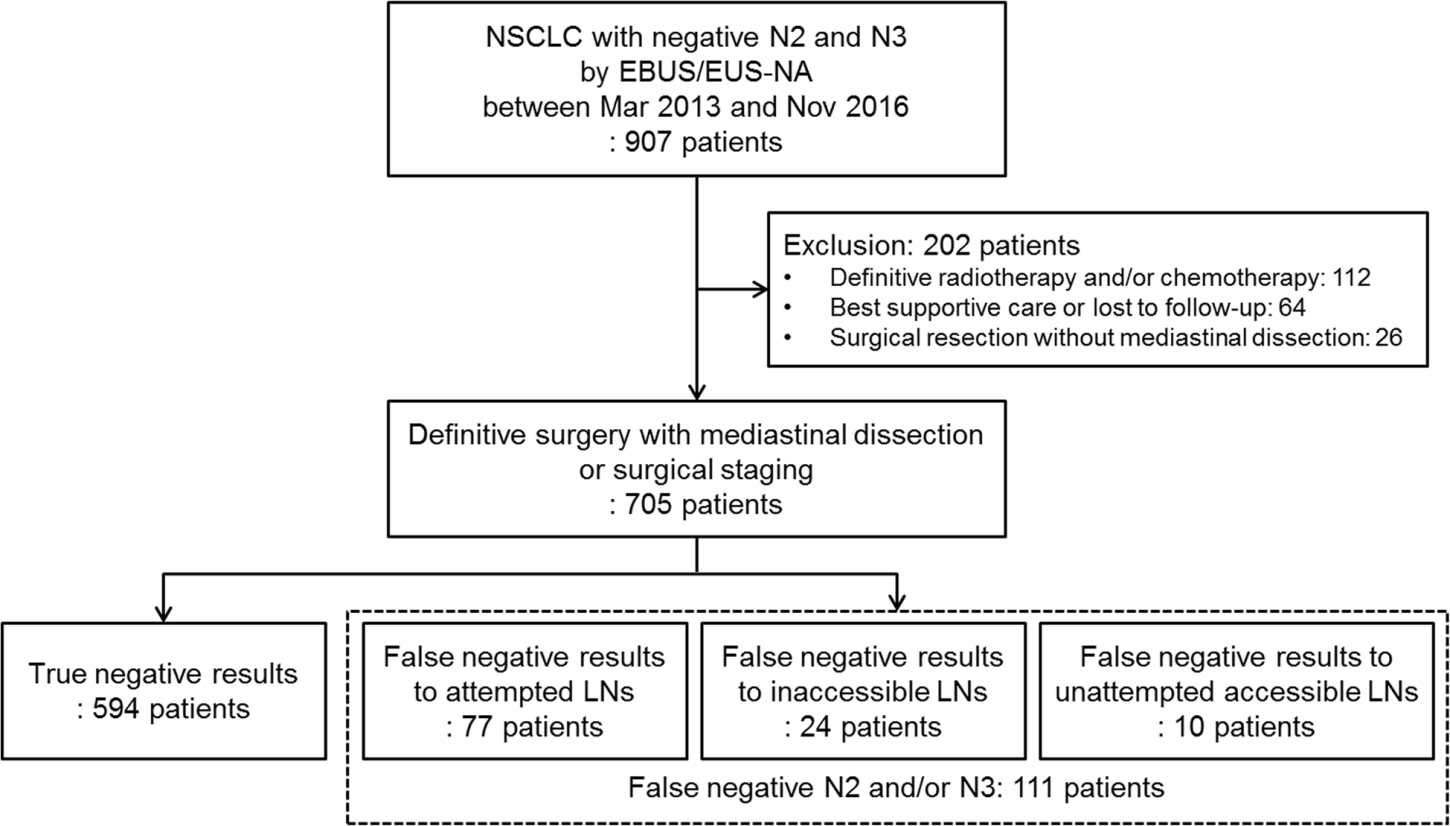
Flow chart. Of 705 enrolled patients, 697 patients underwent definitive surgery with mediastinal dissection, and 8 patients underwent surgical staging before definitive surgery because they were suspected to have mediastinal metastasis even after confirmation of negative results of EBUS/EUS-NA. NSCLC, non-small cell lung cancer; EBUS/EUS-NA, endobronchial ultrasonography and esophageal ultrasonography with needle aspiration; LN, lymph node

We identified 907 patients with NSCLC who had negative results to N2 and N3 by EBUS/EUS-NA (Fig. 1). Among these patients, those who did not undergo definitive surgery with mediastinal dissection or surgical staging were excluded as follows: 112 patients who received definitive radiotherapy and/or chemotherapy, 64 who received best supportive care or were lost to follow-up, and 26 who underwent surgical resection without mediastinal dissection. Of 705 patients who had negative results of EBUS/EUS-NA to N2/3 and underwent definitive surgery with MLND or surgical staging, 111 (15.7%) had positive results to N2 or N3 from MLND and were therefore false negative on EBUS/EUS-NA. These 111 patients were further categorized as 77 (69.4%), 24 (21.6%), and 10 (9.0%) patients with false negative results to attempted LNs, inaccessible LNs, and unattempted accessible LNs, respectively.

### Patient characteristics

We separately investigated patient characteristics of all 705 included patients and of 681 patients after exclusion of those who had inaccessible LNs (Table 1). Among all 705 patients, patients with false negative results of EBUS/EUS-NA were younger and included a higher proportion of females than those with true negative results. However, there were no statistical differences in tumor location, histologic type, duration of procedure, combination of EUS, and number of evaluated lesions by EBUS/EUS-NA between the true and false negative groups. These findings remained even after excluding patients who had inaccessible LNs. Finally, false negative rates of EBUS/EUS-NA to N2/3 varied from 8.3 to 21.4%, but there were no statistical differences according to the operator in either setting (*P* = 0.388 for all patients; *P* = 0.533 excluding patients with inaccessible LNs).

**Table 1:** Baseline characteristics of all included patients and after exclusion of patients with surgically confirmed metastasis to inaccessible lymph nodes

	All patients				Excluding patients with inaccessible LNs			
	Total	True negative	False negative	P	Total	True negative	False negative	P
	(*n* = 705)	(*n* = 594)	(*n* = 111)		(*n* = 681)	(*n* = 594)	(*n* = 87)	
Age, year	65.6 ± 8.7	66.2 ± 8.5	62.4 ± 9.0	< 0.001	65.8 ± 8.6	66.2 ± 8.5	62.7 ± 8.7	0.001
Sex, female	171 (24.3)	135 (22.7)	36 (32.4)	0.029	163 (23.9)	135 (22.7)	28 (32.2)	0.054
Location of primary tumor				0.479				0.331
Right	421 (59.7)	360 (60.6)	61 (55.0)		419 (61.5)	360 (60.6)	59 (67.8)	
Left	278 (39.4)	229 (38.6)	49 (44.1)		256 (37.6)	229 (38.6)	27 (31.0)	
Both	6 (0.9)	5 (0.8)	1 (0.9)		6 (0.9)	5 (0.8)	1 (1.1)	
Histologic type				0.255		277 (46.6)		0.230
Adenocarcinoma	338 (47.9)	277 (46.6)	61 (55.0)		326 (47.9)	273 (46.0)	49 (56.3)	
Squamous cell carcinoma	315 (44.7)	273 (46.0)	42 (37.8)		305 (44.8)	44 (7.4)	32 (36.8)	
Others	52 (7.4)	44 (7.4)	8 (7.2)		50 (7.3)		6 (6.9)	
Duration of procedure, min	19.0 ± 7.7	19.1 ± 7.8	18.1 ± 7.0	0.229	19.0 ± 7.8	19.1 ± 7.8	18.5 ± 7.4	0.353
Combination of EUS	69 (9.8)	55 (9.3)	14 (12.6)	0.275	66 (9.7)	55 (9.3)	11 (12.6)	0.319
Numbers of evaluated lesions								
Total	3.0 ± 1.0	3.0 ± 1.0	2.8 ± 1.0	0.191	3.0 ± 1.0	3.0 ± 1.0	2.9 ± 1.0	0.510
Mediastinal LNs	2.5 ± 0.9	2.5 ± 0.9	2.4 ± 0.9	0.311	2.5 ± 0.9	2.5 ± 0.9	2.4 ± 0.9	0.677
Hilar LNs	0.5 ± 0.7	0.5 ± 0.7	0.4 ± 0.6	0.590	0.4 ± 0.7	0.5 ± 0.7	0.4 ± 0.6	0.733
Lung parenchymal lesions	29 (4.1)	25 (4.2)	4 (3.6)	1.000	28 (4.1)	25 (4.2)	3 (3.4)	1.000
Time interval between EBUS/EUS-NA and surgery, day	19.5 ± 14.6	19.4 ± 14.7	19.6 ± 14.1	0.877	19.2 ± 14.3	19.4 ± 14.7	17.7 ± 10.9	0.307
Operator				0.388				0.533
A	290 (41.1)	240/290 (82.8)	50/290 (17.2)		282 (41.4)	240/282 (85.1)	42/282 (14.9)	
B	219 (31.1)	187/219 (85.4)	32/219 (14.6)		209 (30.7)	187/209 (89.5)	22/209 (10.5)	
C	84 (11.9)	66/84 (78.6)	18/84 (21.4)		79 (11.6)	66/79 (83.5)	13/79 (16.5)	
D	67 (9.5)	60/67 (89.6)	7/67 (10.4)		66 (9.7)	60/66 (90.9)	6/66 (9.1)	
E	33 (4.7)	30/33 (90.9)	3/33 (9.1)		33 (4.8)	30/33 (90.9)	3/33 (9.1)	
F	12 (1.7)	11/12 (91.7)	1/12 (8.3)		12 (1.8)	11/12 (91.7)	1/12 (8.3)	

Data are presented as number (%) or mean ± standard deviation

*LN* lymph node, *EUS* esophageal ultrasonography, *EBUS/EUS-NA* endobronchial ultrasonography and esophageal ultrasonography with needle aspiration

In analysis by operator, there were no statistical differences in patient characteristics of age, sex, tumor location, and histologic type across operators A to F (Additional file 1: Table S1). However, there were significant differences in procedure-related factors of procedure time (range from 16.7 ± 5.5 min for operator C to 34.2 ± 15.2 min for operator E, *P* < 0.001), frequency of EUS combination (1.2% for operator C to 18.2% for operator E, *P* = 0.007), and number of evaluated lesions (2.5 ± 0.6 for operator D to 3.7 ± 1.1 for operator E, *P* < 0.001). These findings persisted after excluding patients who had inaccessible LNs (Additional file 1: Table S2).

### Lymph node characteristics

We separately investigated LN characteristics of 1737 attempted LNs and all 1747 LNs including unattempted accessible LNs (Table 2). There were 10 unattempted accessible LNs with false negative results (station 7 [*n* = 5], 4R [*n* = 4], and 2R [*n* = 1]), which were all less than 5 mm in short-axis on the CT image. Of 1737 attempted LNs, 78 (4.5%) had false negative results of EBUS/EUS-NA. False negative rates were low in station 4 L (5/408, 1.2%); average in stations 2R (4/118, 3.4%), 7 (33/617, 5.3%), and 4R (33/558, 5.9%); and high in stations 5 (2/12, 16.7%) and 8 (1/8, 12.5%). LNs showing false negative results tended to be larger in the short axis. Finally, false negative rates of EBUS/EUS-NA to N2/3 varied from 2.5 to 7.5%, but there were no statistical differences according to the operator in either setting (*P* = 0.250 for attempted LNs; *P* = 0.409 including unattempted accessible LNs).

**Table 2:** Baseline characteristics of attempted lymph nodes only and after inclusion of unattempted accessible lymph nodes

	Attempted LNs				Including unattempted accessible LNs			
	Total	True negative	False negative	P	Total	True negative	False negative	P
	(*n* = 1737)	(*n* = 1659)	(*n* = 78)		(*n* = 1747)	(*n* = 1659)	(*n* = 88)	
Nodal station				0.004				0.001
7	617 (35.5)	584/617 (94.7)	33/617 (5.3)		622 (35.6)	584/622 (93.9)	38/622 (6.1)	
4R	558 (32.1)	525/558 (94.1)	33/558 (5.9)		562 (32.2)	525/562 (93.4)	37/562 (6.6)	
4L	408 (23.5)	403/408 (98.8)	5/408 (1.2)		408 (23.4)	403/408 (98.8)	5/408 (1.2)	
2R	118 (6.8)	114 (96.6)	4/118 (3.4)		119 (6.8)	114/119 (95.8)	5/119 (4.2)	
5	12 (0.7)	10 (83.3)	2/12 (16.7)		12 (0.7)	10/12 (83.3)	2/12 (16.7)	
8	8 (0.5)	7 (87.5)	1/8 (12.5)		8 (0.5)	7/8 (87.5)	1/8 (12.5)	
1R	6 (0.3)	6/6 (100)	0/6 (0)		6 (0.3)	6/6 (100)	0	
9	5 (0.3)	5/5 (100)	0/5 (0)		5 (0.3)	5/5 (100)	0	
3	3 (0.2)	3/3 (100)	0/3 (0)		3 (0.2)	3/3 (100)	0	
2L	2 (0.1)	2/2 (100)	0/2 (0)		2 (0.1)	2/2 (100)	0	
Node size, mm								
Short axis	7.9 ± 2.8	7.8 ± 2.8	8.6 ± 3.5	0.065	7.9 ± 2.8	7.8 ± 2.8	8.6 ± 3.4	0.058
Long axis	12.0 ± 4.9	12.0 ± 4.9	13.1 ± 5.7	0.084	12.0 ± 4.9	12.0 ± 4.9	13.0 ± 5.5	0.058
Number of aspiration per node	1.6 ± 0.7	1.6 ± 0.7	1.7 ± 0.8	0.142	1.6 ± 0.7	1.6 ± 0.7	1.6 ± 1.0	0.474
Obtained core tissue per node	1.4 ± 0.6	1.3 ± 0.6	1.5 ± 0.6	0.017	1.3 ± 0.6	1.3 ± 0.6	1.3 ± 0.7	0.886
Operator				0.250				0.409
A	787 (45.3)	747/787 (94.9)	40/787 (5.1)		791 (45.3)	747/791 (94.4)	44/791 (5.6)	
B	483 (27.8)	467/483 (96.7)	16/483 (3.3)		487 (27.9)	467/487 (95.9)	20/487 (4.1)	
C	185 (10.7)	172/185 (93.0)	13/185 (7.0)		186 (10.6)	172/186 (92.5)	14/186 (7.5)	
D	162 (9.3)	158/162 (97.5)	4/162 (2.5)		163 (9.3)	158/163 (96.9)	5/163 (3.1)	
E	92 (5.3)	88/92 (95.7)	4/92 (4.3)		92 (5.3)	88/92 (95.7)	4/92 (4.3)	
F	28 (1.6)	27/28 (96.4)	1/28 (3.6)		28 (1.6)	27/28 (96.4)	1/28 (3.6)	

Data are presented as number (%) or mean ± standard deviation

*LN* lymph node, *EUS* esophageal ultrasonography

In analysis by operator, there were no statistical differences in the proportion of examined LN stations across operators A to F (Additional file 1: Table S3). However, there were significant differences in the short axis of LN (ranging from 6.9 ± 2.5 mm for operator B to 9.0 ± 2.1 mm for operator F, *P* < 0.001) and procedure-related factors such as number of punctures per node (1.4 ± 0.5 for operator D to 2.0 ± 1.1 for operator E, *P* < 0.001) and obtained core tissue per node (1.2 ± 0.5 for operator D to 1.8 ± 0.8 for operator E, *P* < 0.001). These findings persisted after inclusion of unattempted accessible LNs (Additional file 1: Table S4).

### False negative rate according to operator

**Fig. 2:**
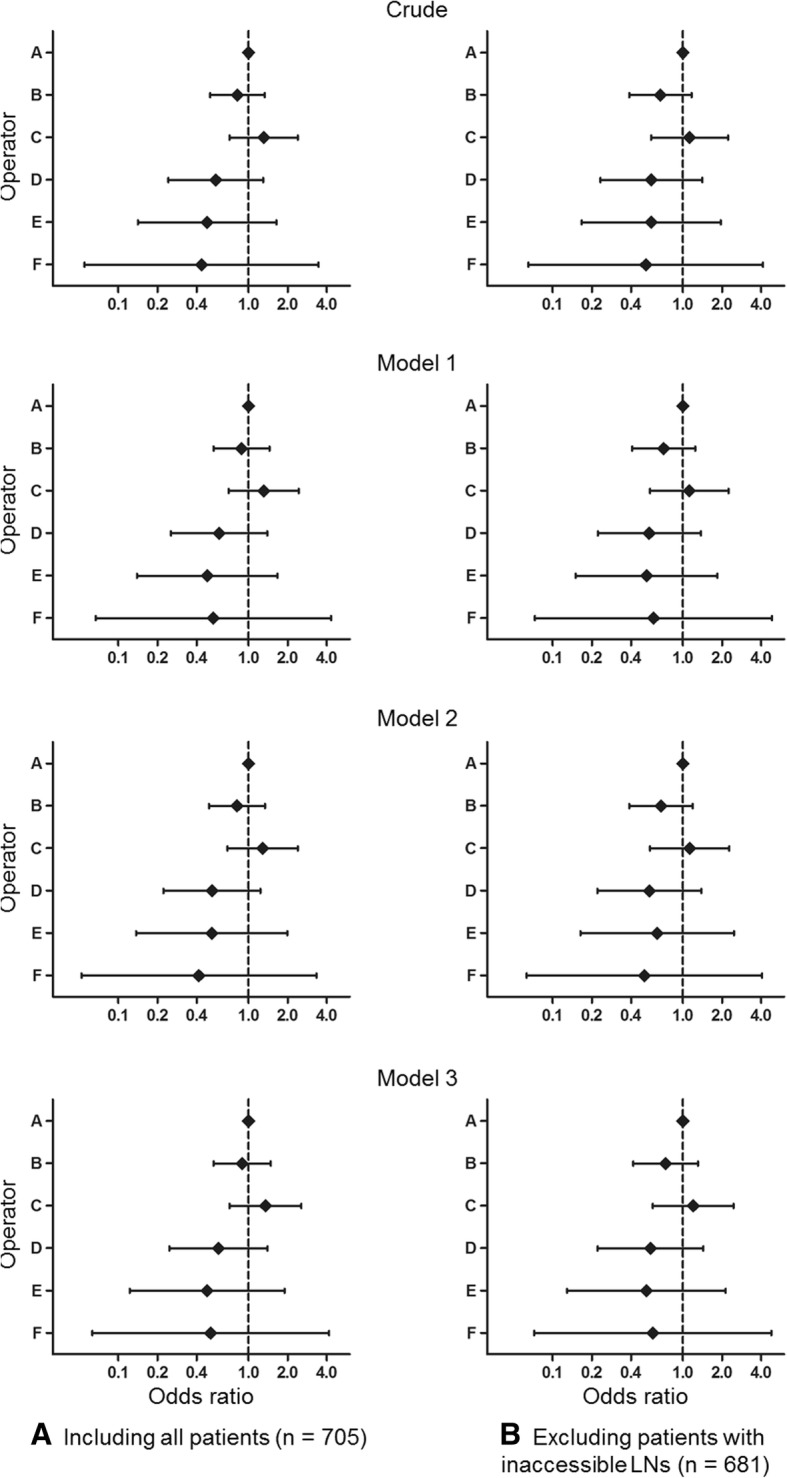
We separately investigated odds ratio of false negative results for (**a**) all 705 included patients and (**b**) 681 patients after exclusion of those who had inaccessible lymph nodes. Odds ratio of false negative results according to operator when analyzing by patient, with operator A as a reference (Details are on Additional file 1: Table S5). Model 1: adjusted for patient character such as age, sex, location and histologic pattern of tumor; Model 2: adjusted for contents of procedure such as duration of procedure, combination of EUS, and number of evaluated lesions; Model 3: adjusted for both patient character and contents of procedure. EUS = esophageal ultrasound

**Fig. 3:**
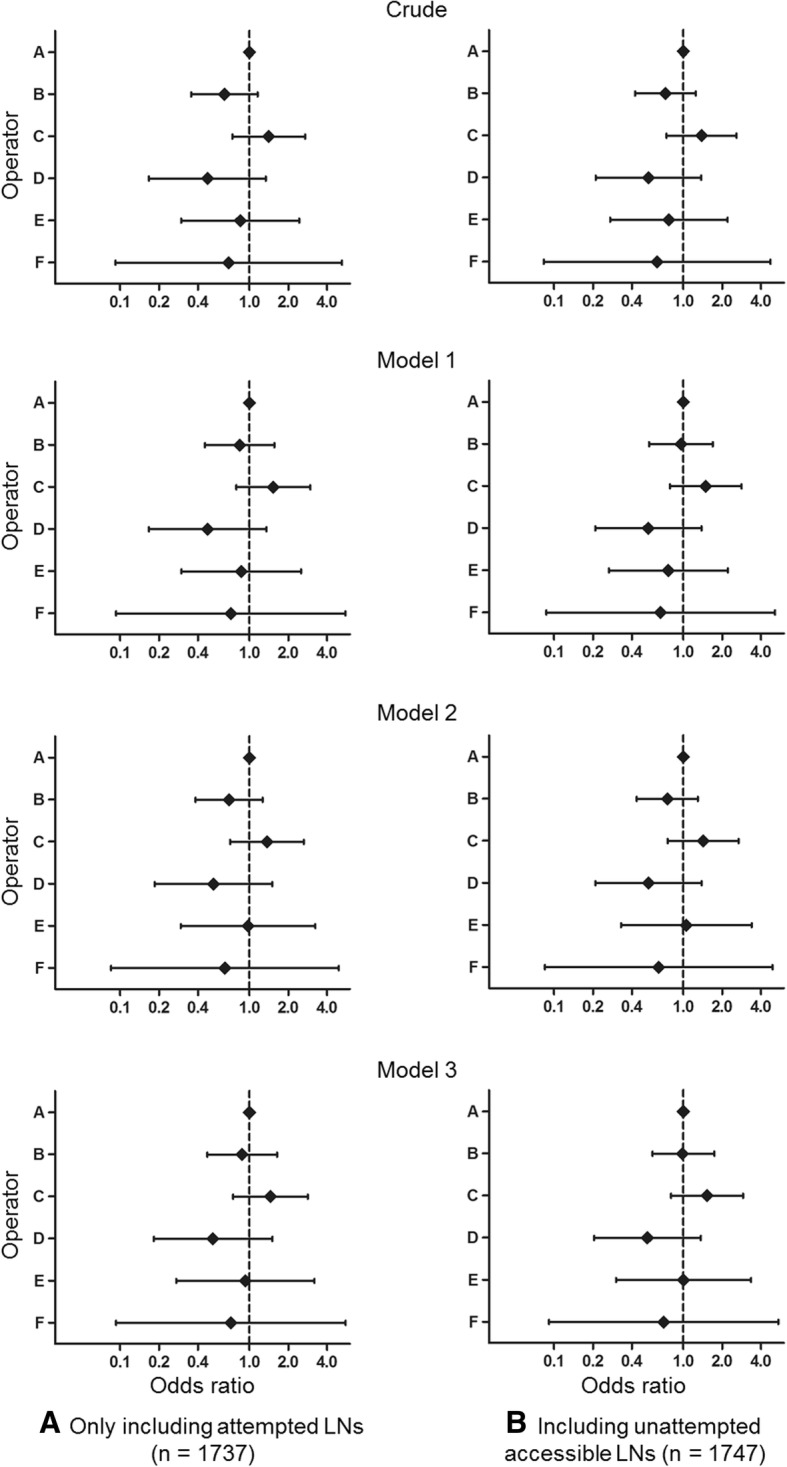
We separately investigated odds ratio of false negative results for (**a**) 1737 attempted lymph nodes and (**b**) all 1747 lymph nodes including unattempted accessible lymph nodes. Odds ratio of false negative results according to operator when analyzing by lymph node, with operator A as a reference (Details are on Additional file 1: Table S6). Model 1: adjusted for patient character such as age, sex, location and histologic pattern of tumor; Model 2: adjusted for contents of procedure such as duration of procedure, combination of EUS, nodal size (short axis), number of aspiration per node, and number of obtained core tissue per node; Model 3: adjusted for both patient character and contents of procedure. EUS = esophageal ultrasound

We calculated the odds ratios for false negative rate by patient (Fig. 2 and details in Additional file 1: Table S5) and LN (Fig. 3 and details in Additional file 1: Table S6) according to operator using operator A as a reference. There were no statistically significant differences in the odds ratios for false negative rate according to operator across the crude model to model 1 (adjusted for patient characteristics), model 2 (adjusted for contents of procedure), and model 3 (adjusted for both patient characteristics and contents of procedure) when analyzing by patient and by LN.

**Fig. 4:**
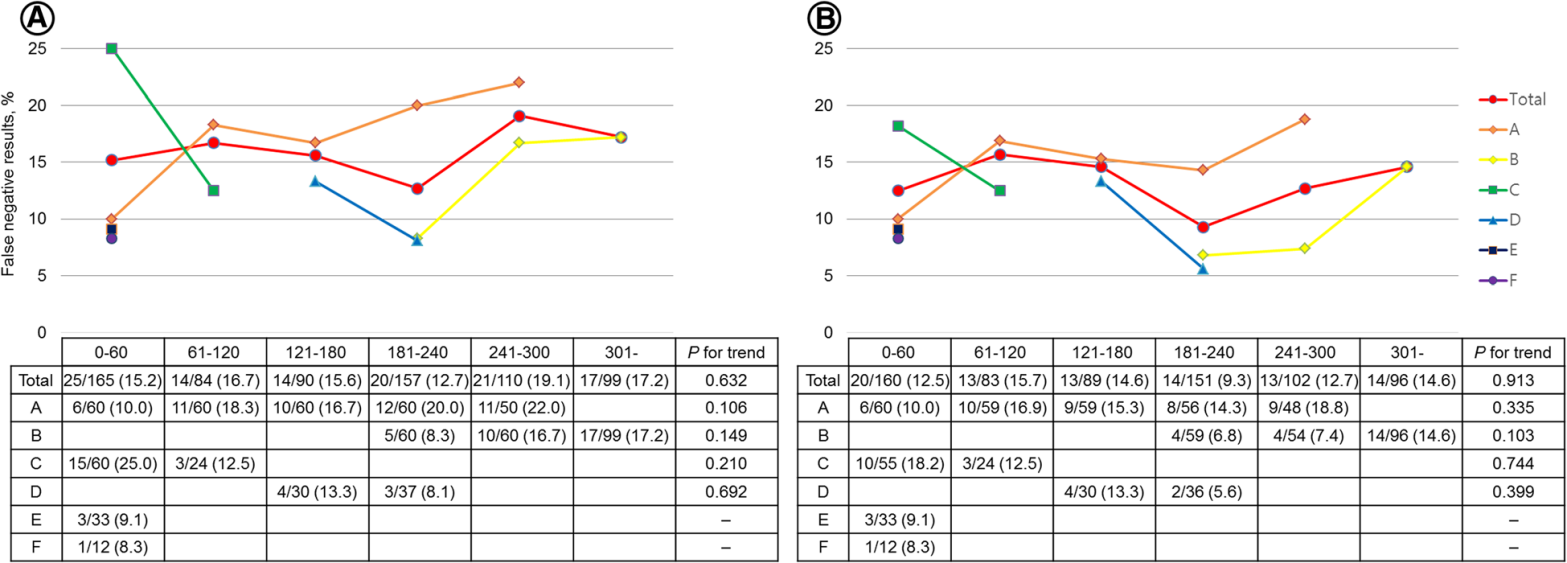
Change in false negative rates according to accumulation of operator experience. Experience of each operator for EBUS/EUS-NA is shown in Additional file 1: Figure S1. There were no statistically significant trends in the change of false negative rates when analyzed for all 705 patients (**a**) and for 681 patients excluding the 24 patients who had false negative results to inaccessible lymph nodes (**b**)

We also investigated whether the false negative rate changed according to the operator’s experience for all 705 patients (Fig. 4a) and for 681 patients after excluding 24 patients who had inaccessible LNs (Fig. 4b). There were no statistically significant trends in false negative rates according to experience for all operators.

## Discussion

We retrospectively identified 705 patients with NSCLC who had negative N2/3 by EBUS/EUS-NA performed by six operators and subsequently underwent MLND. This study population included 111 (15.7%) false negative cases. In our institution, an operator can independently perform EBUS/EUS-NA without a supervisor after at least 200 cases of observation and assistance over a period of 6 months. Nevertheless, procedure-related factors such as duration of procedure, frequency of combination with EUS, number of evaluated lesions, node size, and obtained core tissue per node varied among operators. There were no statistically significant differences in baseline characteristics of age, sex, location of primary tumor, histologic type, and evaluated nodal station according to operator. False negative rates to N2/3 LNs according to operator were not statistically different. In addition, there was no trend in the change of false negative rate according to accumulation of operator experience. Therefore, although there are differences in procedure style according to operator, there appears to be no difference in false negative rates after the operator has completed sufficient training.

In the present study, the rate of false negative results on N2/3 LNs by EBUS/EUS-NA was 111/705 (15.7%). Among the patients with false negative results, 24/111 (21.6%) had false negative results to inaccessible LNs, and these rates were similar to those reported in previous studies [9, 10]. Previous studies have identified several factors associated with false negative results of EBUS/EUS-NA [9, 10, 17], including primary tumor location (central or left lung), abnormal findings of mediastinal LNs on CT and/or PET, inadequacy of the sampling, internal necrosis in the LNs, and rare cancer types. Although we did not perform multivariate analysis to identify the independent patient and tumor characteristics associated with false negative results, young age, female gender, inaccessible stations by EBUS, and larger size of LNs had a trend for higher false negative rates (Tables 1 and 2).

There are several studies on the learning curve of EBUS/EUS-NA. Some studies have concluded that optimal accuracy of the procedure can be achieved after a relatively short learning curve (about 10 procedures) [12, 18]. However, another study demonstrated that the minimal diagnostic yield was achieved with 60 procedures, but optimal results were obtained after 100 procedures [19]. In another study, significant variation was seen in the learning curves of individual operators, with ongoing improvement in EBUS/EUS-NA skill even after 200 clinical cases [7]. In the present study, there was no difference in the false negative rate among the operators despite differences in procedure style. The differences in interpretation of the learning curve for operators between these studies may be explained by the fact that most of the previous studies involved EBUS novices, whereas our study was conducted on operators with experience of observation and assistance for least 200 cases. Recent research showed that simulation-based training using an assessment tool is effective for improving the EBUS performance metrics [11, 20, 21]. However, no study has evaluated whether the skills demonstrated on a simulation assessment are transferred to an improvement in clinical skills as performed in patients [22]. We think that sufficient clinical experience, even indirect experience such as observation and assistance, is necessary to increase the diagnostic performance in real clinical practice.

There are several limitations of this study that affect the generalizability of our results. First, this is a retrospective study performed in only one hospital, and EBUS/EUS-NA was performed without general anesthesia or ROSE. However, we evaluated cases performed by six operators with various degrees of experience and different operator characteristics such as duration of procedure, frequency of combination with EUS, number of evaluated lesions, node size, and obtained core tissue per node. We think that the variation in operator characteristics may overcome some of the limitations associated with single-institution research. Second, we were not able to evaluate the complication rate according to each operator and experience accumulation. Third, we have no data on false negative rates to N1 LNs. Although differentiation between N0/1 and N2/3 is the most important factor in N-staging of NSCLC because it greatly influences the decision of treatment policy, some recent papers report that differentiation between N0 and N1 is also important because it may influence delicate decisions such as neoadjuvant clinical trials for stage II NSCLC, selective MLND, and selective limited resection [23, 24]. Therefore, further research is needed to evaluate the false negative rates to N1 LNs. Finally, we did not confirm whether 200 cases of observation and assistance during 6 months is the optimum training for the EBUS/EUS-NA procedure.

## Conclusions

In conclusion, if the operator has sufficient experience of bronchoscopy and at least 6 months of experience of observation and assistance in EBUS/EUS-NA, the false negative rate to N2/3 LNs might be not affected by the operator. In addition, false negative rates to N2/3 LNs may not be reduced by additional experience. However, further studies are needed to evaluate the minimum amount of observation and assistance required before performing the independent EBUS/EUS-NA procedure.

## Additional file

Additional file 1: **Figure S1**. Experience of each operator with EBUS/EUS-NA. **Table S1.** Baseline characteristics, contents of the procedure, and false negative rate of the total 705 patients analyzed by each operator. **Table S2.** Baseline characteristics, contents of the procedure, and false negative rate of 681 patients, excluding 24 patients who had false negative result from inaccessible LNs, analyzed by each operator. **Table S3.** Baseline characteristics, contents of the procedure, and false negative rate of the total 1,737 attempted LNs analyzed by each operator. **Table S4.** Baseline characteristics, contents of the procedure, and false negative rate of 1,747 attempted LNs (including 10 unattempted accessible LNs) analyzed by each operator. **Table S5.** Odds ratios for false negative results by operator analyzed by patient with operator A as reference. **Table S6.** Odds ratios for false negative results by operator analyzed by LN with operator A as reference. (ZIP 91 kb)

## Abbreviations

CT: Computed tomography
EBUS: Endobronchial ultrasound
EBUS/EUS-NA: Endosonography (EBUS and EUS) with needle aspiration
EUS: Esophageal ultrasound
IASLC: International Association for the Study of Lung Cancer
LN: Lymph node
MLND: Mediastinal lymph node dissection
NSCLC: Non-small cell lung cancer
PET: Positron emission tomography
ROSE: Rapid on-site cytopathological examination
TNM: Tumor-node-metastasis
