# Correction to: Are there differences among operators in false-negative rates of endosonography with needle aspiration for mediastinal nodal staging of non-small cell lung cancer?

**DOI:** 10.1186/s12890-019-0805-y

**Published:** 2019-03-25

**Authors:** Sukyeon Kim, Beomsu Shin, Hyun Lee, Jick Hwan Ha, Kyungjong Lee, Sang-Won Um, Hojoong Kim, Byeong-Ho Jeong

**Affiliations:** 1 Division of Pulmonary Medicine, Department of Internal medicine, Hangang Sacred Heart Hospital, Hallym University School of Medicine, Seoul, Republic of Korea; 2 Department of Internal Medicine, Yonsei University Wonju College of Medicine, Wonju, Republic of Korea; 3 Division of Pulmonary Medicine and Allergy, Department of Internal Medicine, Hanyang University College of Medicine, Seoul, Republic of Korea; 4 Division of Pulmonology, Critical Care and Sleep Medicine, Department of Internal Medicine, College of Medicine, The Catholic University of Korea, Seoul, Republic of Korea; 5 Division of Pulmonary and Critical Care Medicine, Department of Medicine, Samsung Medical Center, Sungkyunkwan University School of Medicine, Irwon-ro 81, Gangnam-gu, Seoul, 06351 Republic of Korea

**Correction to: BMC Pulmonary Medicine (2018) 19:14**

**DOI: 10.1186/s12890-018-0774-6**

Following publication of the original article [1], the author flagged that the ‘True negative’ values given in Table 1, for ‘Excluding patients with inaccessible LNs’, were wrong for ‘Adenocarcinoma’, ‘Squamous cell carcinoma’ and ‘Others’.

The (incorrect) values previously given were 273 (46.0) for ‘Adenocarcinoma’, 44 (7.4) for ‘Squamous cell carcinoma’, and a blank value for ‘Others’.

Please be advised that **the correct values are**:

277 (46.6) for ‘Adenocarcinoma’.

273 (46.0) for ‘Squamous cell carcinoma’.

44 (7.4) for ‘Other’.

The original article has now been corrected to reflect this.

The publisher apologizes for this error.
