# Supplementary material for: Are there differences among operators in false-negative rates of endosonography with needle aspiration for mediastinal nodal staging of non-small cell lung cancer?
Source: BMC Pulm Med. 2019 Jan 14;19:14. doi: 10.1186/s12890-018-0774-6 (PMC6332520; doi:10.1186/s12890-018-0774-6)
Supplement: Supplementary file 1 — Figure S1. Experience of each operator with EBUS/EUS-NA. Table S1. Baseline characteristics, contents of the procedure, and false negative rate of the total 705 patients analyzed by each operator. Table S2. Baseline characteristics, contents of the procedure, and false negative rate of 681 patients, excluding 24 patients who had false negative result from inaccessible LNs, analyzed by each operator. Table S3. Baseline characteristics, contents of the procedure, and false negative rate of the total 1,737 attempted LNs analyzed by each operator. Table S4. Baseline characteristics, contents of the procedure, and false negative rate of 1,747 attempted LNs (including 10 unattempted accessible LNs) analyzed by each operator. Table S5. Odds ratios for false negative results by operator analyzed by patient with operator A as reference. Table S6. Odds ratios for false negative results by operator analyzed by LN with operator A as reference. (ZIP 91 kb) [file 12890_2018_774_MOESM1_ESM.zip › BMC_PM Online suppl marked 181022R1.docx]

**Online Supplement**

**Are there differences among operators in false-negative rates of endosonography with needle aspiration for mediastinal nodal staging of non-small cell lung cancer?**

Sukyeon Kim, MD^1^; Beomsu Shin, MD^2^; Hyun Lee, MD^3^; [Jick Hwan Ha](https://www.kjccm.org/articles/search_result.php?term=author&f_name=Jick%20Hwan&l_name=Ha), MD^4^; Kyung Jong Lee, MD^5^; Sang-Won Um, MD^5^; Hojoong Kim, MD^5^; Byeong-Ho Jeong, MD^5^

Division of Pulmonary Medicine, Department of Internal medicine, Hangang Sacred Heart Hospital, Hallym University School of Medicine, Seoul, Republic of Korea^1^

Department of Internal Medicine, Yonsei University Wonju College of Medicine, Wonju, Republic of Korea^2^

Division of Pulmonary Medicine and Allergy, Department of Internal Medicine, Hanyang University College of Medicine, Seoul, Republic of Korea^3^

Division of Pulmonology, Critical Care and Sleep Medicine, Department of Internal Medicine, College of Medicine, The Catholic University of Korea, Seoul, Republic of Korea^4^

Division of Pulmonary and Critical Care Medicine, Department of Medicine, Samsung Medical Center, Sungkyunkwan University School of Medicine, Seoul, Republic of Korea^5^

**Corresponding author**: Byeong-Ho Jeong, MD, PhD

Division of Pulmonary and Critical Care Medicine, Department of Medicine, Samsung Medical Center, Sungkyunkwan University School of Medicine, Irwon-ro 81, Gangnam-gu, Seoul 06351, South Korea.

Tel: (+82) 2 3410 3429, Fax: (+82) 2 3410 3849

E-mail: myacousticlung@gmail.com

**e-Figure 1.** Experience of each operator with EBUS/EUS-NA. The denominator is the total number of EBUS/EUS-NA procedures for each operator in their career period, and the numerator is the number of EBUS/EUS-NA procedures of each operator included in this study. Operator A participated for the entire period of this study and performed 1,228 cases of EBUS/EUS-NA. Of these cases, 290 cases with negative results of EBUS/EUS-NA for nodal staging of NSCLC patients were analyzed. Operators B and D already had many experiences with the EBUS/EUS-NA procedure at the start of this study and participated for a certain period of the study. And, operator B and D performed 1,072 and 279 cases of EBUS/EUS-NA during the study period, respectively. Of these cases, 219 and 67 cases with negative results of EBUS/EUS-NA for nodal staging of NSCLC patients were analyzed for operator B and D, respectively. Operators C, E, and F started performing the EBUS/EUS-NA procedure in the middle of the study, and they performed 368, 143, and 84 cases of EBUS/EUS-NA during the study period, respectively. Of these cases, 84, 33, and 12 cases with negative results of EBUS/EUS-NA for nodal staging of NSCLC patients were analyzed for operator C, E, and F, respectively.

EBUS/EUS-NA = endobronchial ultrasound and esophageal ultrasound with needle aspiration, NSCLC = non-small cell lung cancer.

**e-Table 1.** Baseline characteristics, contents of the procedure, and false negative rate of the total 705 patients analyzed by each operator.

|  | Total  (n = 705) | A  (n = 290) | B  (n = 219) | C  (n = 84) | D  (n = 67) | E  (n = 33) | F  (n = 12) | *P* |
| --- | --- | --- | --- | --- | --- | --- | --- | --- |
| Age, year | 65.6 ± 8.7 | 64.9 ± 9.3 | 66.4 ± 8.5 | 65.1 ± 8.2 | 65.3 ± 7.2 | 66.4 ± 8.5 | 69.9 ± 7.4 | 0.185 |
| Sex, female | 171 (24.3) | 66 (22.8) | 48 (21.9) | 26 (31.0) | 15 (22.4) | 12 (36.4) | 4 (33.3) | 0.256 |
| Location of primary tumor |  |  |  |  |  |  |  |  |
| Right  Left  Both | 421 (59.7)  278 (39.4)  6 (0.9) | 169 (58.3)  119 (41.0)  2 (0.7) | 128 (58.4)  91 (41.6)  0 | 46 (54.8)  37 (44.0)  1 (1.2) | 48 (71.6)  18 (26.9)  1 (1.5) | 22 (66.7)  9 (27.3)  2 (6.1) | 8 (66.7)  4 (33.3)  0 | 0.294  0.145  0.035 |
| Histologic type  Adenocarcinoma  Squamous cell carcinoma  Others | 338 (47.9)  315 (44.7)  52 (7.4) | 139 (47.9)  128 (44.1)  23 (7.9) | 98 (44.7)  110 (50.2)  11 (5.0) | 40 (47.6)  33 (39.3)  11 (13.1) | 34 (50.7)  29 (43.3)  4 (6.0) | 21 (63.6)  9 (27.3)  3 (9.1) | 6 (50.0)  6 (50.0)  0 | 0.495  0.157  0.238 |
| Duration of procedure, min | 19.0 ± 7.7 | 17.7 ± 6.0^e^ | 19.2 ± 6.7^c,e^ | 16.7 ± 5.5^b,e^ | 18.6 ± 6.3^e^ | 34.2 ± 15.2^a,b,c,d,f^ | 20.8 ± 5.2^e^ | <0.001 |
| Combined EUS | 69 (9.8) | 35 (12.1)^c^ | 18 (8.2)^c^ | 1 (1.2)^a,b^ | 7 (10.4) | 6 (18.2) | 2 (16.7) | 0.007 |
| Numbers of evaluated lesions  Total  Mediastinal LNs  Hilar LNs  Lung parenchymal lesions | 3.0 ± 1.0  2.5 ± 0.9  0.5 ± 0.7  29 (4.1) | 3.1 ± 1.0^c,d^  2.7 ± 0.8^b,c,d^  0.4 ± 0.6^b,d,e^  13 (4.5) | 2.9 ± 1.0^c,d,e^  2.2 ± 0.9^a,e^  0.6 ± 0.7^a,c,d^  11 (5.0) | 2.6 ± 0.8^a,b,e^  2.2 ± 0.6^a,e^  0.3 ± 0.5^b,d,e^  1 (1.2) | 2.5 ± 0.6^a,b,e^  2.4 ± 0.7^a^  0.1 ± 0.33^a,b,c,e^  1 (1.5) | 3.7 ± 1.1^b,c,d^  2.8 ± 0.9^b,c^  0.9 ± 0.8^a,c,d^  2 (6.1) | 2.9 ± 0.8  2.1 ± 0.8  0.8 ± 0.8  1 (8.3) | <0.001  <0.001  <0.001  0.361 |
| Time interval between EBUS/EUS-NA and surgery, day | 19.5 ± 14.6 | 18.8 ± 11.6 | 19.1 ± 13.4 | 20.4 ± 16.1 | 21.4 ± 26.5 | 20.4 ± 10.5 | 21.6 ± 7.7 | 0.663 |
| False negative | 111 (15.7) | 50 (17.2) | 32 (14.6) | 18 (21.4) | 7 (10.4) | 3 (9.1) | 1 (8.3) | 0.388 |

^a,b,c,d,e,f^ In case of statistically significant differences (*P* < 0.05) by post-hoc analysis, the corresponding operator were marked as lowercase letters.

EUS = esophageal ultrasound, LN = lymph node, EBUS/EUS-NA = endobronchial ultrasonography and esophageal ultrasonography with needle aspiration.

**e-Table 2.** Baseline characteristics, contents of the procedure, and false negative rate of 681 patients, excluding 24 patients who had false negative result from inaccessible LNs, analyzed by each operator.

|  | Total  (n = 681) | A  (n = 282) | B  (n = 209) | C  (n = 79) | D  (n = 66) | E  (n = 33) | F  (n = 12) | *P* |
| --- | --- | --- | --- | --- | --- | --- | --- | --- |
| Age, year | 65.8 ± 8.6 | 65.1 ± 9.1 | 66.5 ± 8.5 | 65.3 ± 8.2 | 65.4 ± 7.3 | 66.4 ± 8.5 | 69.9 ± 7.4 | 0.256 |
| Sex, female | 163 (23.9) | 65 (23.0) | 43 (20.6) | 24 (30.4) | 15 (22.7) | 12 (36.4) | 4 (33.3) | 0.229 |
| Location of primary tumor |  |  |  |  |  |  |  |  |
| Right  Left  Both | 419 (61.5)  256 (37.6)  6 (0.9) | 168 (59.6)  112 (39.7)  2 (0.7) | 127 (60.8)  82 (39.2)  0 | 46 (58.2)  32 (40.5)  1 (1.3) | 48 (72.7)  17 (25.8)  1 (1.5) | 22 (66.7)  9 (27.3)  2 (6.1) | 8 (66.7)  4 (33.3)  0 | 0.431  0.249  0.039 |
| Histologic type  Adenocarcinoma  Squamous cell carcinoma  Others | 326 (47.9)  305 (44.8)  50 (7.3) | 135 (47.9)  124 (44.0)  23 (8.2) | 93 (44.5)  105 (50.2)  11 (5.3) | 38 (48.1)  32 (40.5)  9 (11.4) | 33 (40.0)  29 (43.9)  4 (6.1) | 21 (63.6)  9 (27.3)  3 (9.1) | 6 (50.0)  6 (50.0)  0 | 0.496  0.191  0.481 |
| Duration of procedure, min | 19.0 ± 7.8 | 17.6 ± 6.0^e^ | 19.3 ± 6.8^c,e^ | 16.6 ± 5.6^b,e^ | 18.7 ± 6.3^e^ | 34.2 ± 15.2^a,b,c,d,f^ | 20.8 ± 5.2^e^ | <0.001 |
| Combined EUS | 66 (9.7) | 33 (11.7)^c^ | 17 (8.1)^c^ | 1 (1.3)^a,b^ | 7 (10.6) | 6 (18.2) | 2 (16.7) | 0.012 |
| Numbers of evaluated lesions  Total  Mediastinal LNs  Hilar LNs  Lung parenchymal lesions | 3.0 ± 1.0  2.5 ± 0.9  0.4 ± 0.7  28 (4.1) | 3.1 ± 1.0^c,d^  2.7 ± 0.9^b,c,d^  0.4 ± 0.6^b,d,e^  13 (4.6) | 2.9 ± 1.0^c,d,e^  2.2 ± 0.9^a,e^  0.4 ± 0.7^a,c,d^  10 (4.8) | 2.5 ± 0.7^a,b,e^  2.2 ± 0.6^a,e^  0.3 ± 0.5^b,e^  1 (1.3) | 2.6 ± 0.6^a,b,e^  2.4 ± 0.7^a^  0.1 ± 0.3^a,b,e^  1 (1.5) | 3.7 ± 1.1^b,c,d^  2.8 ± 0.9^b,c^  0.9 ± 0.8^a,c,d^  2 (6.1) | 2.9 ± 0.8  2.1 ± 0.8  0.8 ± 0.8  1 (8.3) | <0.001  <0.001  <0.001  0.410 |
| Time interval between EBUS/EUS-NA and surgery, day | 19.2 ± 14.3 | 18.8 ± 11.7 | 18.5 ± 12.6 | 19.7 ± 14.8 | 21.6 ± 26.7 | 20.4 ± 10.5 | 21.6 ± 7.7 | 0.663 |
| False negative | 87 (12.8) | 42 (14.9) | 22 (10.5) | 13 (16.5) | 6 (9.1) | 3 (9.1) | 1 (8.3) | 0.533 |

^a,b,c,d,e,f^ In case of statistically significant differences (*P* < 0.05) by post-hoc analysis, the corresponding operator were marked as lowercase letter.

EUS = esophageal ultrasound, LN = lymph node, EBUS/EUS-NA = endobronchial ultrasonography and esophageal ultrasonography with needle aspiration.

**e-Table 3.** Baseline characteristics, contents of the procedure, and false negative rate of the total 1,737 attempted LNs analyzed by each operator.

|  | Total  (n = 1737) | A  (n = 787) | B  (n = 483) | C  (n = 185) | D  (n = 162) | E  (n = 92) | F  (n = 28) | *P* |
| --- | --- | --- | --- | --- | --- | --- | --- | --- |
| Nodal station  7  4R  4L  2R  5  8  1R  9  3  2L | 617 (35.5)  558 (32.1)  408 (23.5)  118 (6.8)  12 (0.7)  8 (0.5)  6 (0.3)  5 (0.3)  3 (0.2)  2 (0.1) | 262 (33.3)  254 (32.3)  207 (26.3)  48 (6.1)  8 (1.0)  3 (0.4)  2 (0.3)  0  1 (0.1)  2 (0.3) | 176 (36.4)  149 (30.8)  100 (20.7)  42 (8.7)  3 (0.6)  4 (0.8)  2 (0.4)  5 (1.0)  2 (0.4)  0 | 75 (40.5)  65 (35.1)  35 (18.9)  8 (4.3)  1 (0.5)  0  1 (0.5)  0  0  0 | 61 (37.7)  53 (32.7)  36 (22.2)  12 (7.4)  0  0  0  0  0  0 | 32 (34.8)  26 (28.3)  26 (28.3)  7 (7.6)  0  0  1 (1.1)  0  0  0 | 11 (39.3)  11 (39.3)  4 (14.3)  1 (3.6)  0  1 (3.6)  0  0  0  0 | 0.488  0.784  0.066  0.346  0.850  0.215  0.515  0.050  0.829  0.748 |
| Node size, mm  Short axis  Long axis | 7.9 ± 2.8  12.0 ± 4.9 | 8.3 ± 2.8^b^  13.4 ± 5.1^b,c,e^ | 6.9 ± 2.5^a,c,d,e,f^  9.4 ± 3.7^a,c,d,e,f^ | 7.8 ± 3.1^b,d^  11.1 ± 4.8^a,b,d,f^ | 8.8 ± 2.7^b,c^  13.5 ± 4.7^b,c^ | 7.9 ± 2.2^b^  12.0 ± 3.5^a,b,f^ | 9.0 ± 2.1^b^  15.6 ± 4.7^b,c,e^ | <0.001  <0.001 |
| Number of puncture per node | 1.6 ± 0.7 | 1.7 ± 0.7^d^ | 1.6 ± 0.7^c,d,e^ | 1.7 ± 0.6^b,d^ | 1.4 ± 0.5^a,b,c,e,f^ | 2.0 ± 1.1^b,d^ | 1.9 ± 0.7^e^ | <0.001 |
| Obtained core tissue per node | 1.4 ± 0.6 | 1.3 ± 0.5^c,d,e^ | 1.3 ± 0.6^c,e^ | 1.5 ± 0.5^a,b,d,e^ | 1.2 ± 0.5^a,c,e,f^ | 1.8 ± 0.8^a,b,c,d^ | 1.6 ± 0.5^d^ | <0.001 |
| False negative | 78 (4.5) | 40 (5.1) | 16 (3.3) | 13 (7.0) | 4 (2.5) | 4 (4.3) | 1 (3.6) | 0.250 |

^a,b,c,d,e,f^ In case of statistically significant differences (*P* < 0.05) by post-hoc analysis, the corresponding operator were marked as lowercase letter.

LN = lymph node.

**e-Table 4.** Baseline characteristics, contents of the procedure, and false negative rate of 1,747 attempted LNs (including 10 unattempted accessible LNs) analyzed by each operator.

|  | Total  (n = 1747) | A  (n = 791) | B  (n = 487) | C  (n = 186) | D  (n = 163) | E  (n = 92) | F  (n = 28) | *P* |
| --- | --- | --- | --- | --- | --- | --- | --- | --- |
| Nodal station  7  4R  4L  2R  5  8  1R  9  3  2L | 622 (35.6)  562 (32.2)  408 (23.4)  119 (6.8)  12 (0.7)  8 (0.5)  6 (0.3)  5 (0.3)  3 (0.2)  2 (0.1) | 265 (33.5)  254 (32.1)  207 (26.2)  49 (6.2)  8 (1.0)  3 (0.4)  2 (0.3)  0  1 (0.1)  2 (0.3) | 178 (36.6)  151 (31.0)  100 (20.5)  42 (8.6)  3 (0.6)  4 (0.8)  2 (0.4)  5 (1.0)  2 (0.4)  0 | 75 (40.3)  66 (35.5)  35 (18.8)  8 (4.3)  1 (0.5)  0  1 (0.5)  0  0  0 | 61 (37.4)  54 (33.1)  36 (22.1)  12 (7.4)  0  0  0  0  0  0 | 32 (34.8)  26 (28.3)  26 (28.3)  7 (7.6)  0  0  1 (1.1)  0  0  0 | 11 (39.3)  11 (39.3)  4 (14.3)  1 (3.6)  0  1 (3.6)  0  0  0  0 | 0.557  0.761  0.063  0.381  0.849  0.211  0.498  0.051  0.828  0.747 |
| Node size, mm  Short axis  Long axis | 7.9 ± 2.8  12.0 ± 4.9 | 8.3 ± 2.8^b^  13.3 ± 5.1^b,c,e^ | 6.9 ± 2.5^a,c,d,e,f^  9.5 ± 3.7^a,c,d,e,f^ | 7.8 ± 3.1^b,d^  11.1 ± 4.8^a,b,d,f^ | 8.8 ± 2.7^b,c^  13.5 ± 4.7^b,c^ | 7.9 ± 2.2^b^  12.0 ± 3.5^a,b,f^ | 9.0 ± 2.1^b^  15.6 ± 4.7^b,c,e^ | <0.001  <0.001 |
| Number of puncture per node | 1.6 ± 0.7 | 1.6 ± 0.7^d^ | 1.5 ± 0.7^c,e^ | 1.7 ± 0.6^b,d^ | 1.4 ± 0.5^a,c,e,f^ | 2.0 ± 1.1^b,d^ | 1.9 ± 0.7^d^ | <0.001 |
| Obtained core tissue per node | 1.3 ± 0.6 | 1.3 ± 0.6^d,e^ | 1.3 ± 0.6^c,e^ | 1.5 ± 0.6^b,d,e^ | 1.2 ± 0.5^a,c,e,f^ | 1.8 ± 0.8^a,b,c,d^ | 1.6 ± 0.5^d^ | <0.001 |
| False negative | 88 (5.0) | 44 (5.6) | 20 (4.1) | 14 (7.5) | 5 (3.1) | 4 (4.3) | 1 (3.6) | 0.409 |

^a,b,c,d,e,f^ In case of statistically significant differences (*P* < 0.05) by post-hoc analysis, the corresponding operator were marked as lowercase letter.

LN = lymph node.

**e-Table 5.** Odds ratios for false negative results by operator analyzed by patient with operator A as reference.

|  | Including all patients (n = 705) | | | | Excluding patients with inaccessible LNs (n = 681) | | | |
| --- | --- | --- | --- | --- | --- | --- | --- | --- |
|  | Crude | Model 1 | Model 2 | Model 3 | Crude | Model 1 | Model 2 | Model 3 |
| A | Ref | Ref | Ref | Ref | Ref | Ref | Ref | Ref |
| B | 0.821 (0.507-1.332) | 0.884 (0.539-1.448) | 0.816 (0.498-1.340) | 0.893 (0.539-1.481) | 0.672 (0.388-1.165) | 0.711 (0.407-1.245) | 0.679 (0.387-1.192) | 0.737 (0.416-1.307) |
| C | 1.309 (0.716-2.394) | 1.308 (0.704-2.429) | 1.285 (0.691-2.392) | 1.351 (0.715-2.550) | 1.126 (0.571-2.220) | 1.121 (0.561-2.240) | 1.127 (0.560-2.268) | 1.201 (0.588-2.452) |
| D | 0.560 (0.242-1.297) | 0.595 (0.254-1.392) | 0.524 (0.222-1.232) | 0.587 (0.247-1.397) | 0.571 (0.232-1.407) | 0.551 (0.222-1.372) | 0.553 (0.220-1.387) | 0.563 (0.221-1.431) |
| E | 0.480 (0.141-1.634) | 0.482 (0.139-1.675) | 0.523 (0.137-1.988) | 0.481 (0.122-1.896) | 0.571 (0.167-1.957) | 0.525 (0.150-1.841) | 0.635 (0.163-2.469) | 0.526 (0.129-2.134) |
| F | 0.436 (0.055-3.457) | 0.536 (0.067-4.321) | 0.416 (0.052-3.328) | 0.511 (0.063-4.155) | 0.519 (0.065-4.130) | 0.595 (0.073-4.826) | 0.506 (0.063-4.063) | 0.587 (0.072-4.792) |
| *P* value | 0.346 | 0.487 | 0.384 | 0.494 | 0.494 | 0.550 | 0.551 | 0.596 |

Model 1: adjusted for patient character such as age, sex, location and histologic pattern of tumor.

Model 2: adjusted for contents of procedure such as duration of procedure, combination of EUS, and number of evaluated lesions.

Model 3: adjusted for both patient character and contents of procedure.

LN = lymph node; EUS = esophageal ultrasound.

**e-Table 6.** Odds ratios for false negative results by operator analyzed by LN with operator A as reference.

|  | Only including attempted LNs (n = 1737) | | | | Including unattempted accessible LNs (n = 1747) | | | |
| --- | --- | --- | --- | --- | --- | --- | --- | --- |
|  | Crude | Model 1 | Model 2 | Model 3 | Crude | Model 1 | Model 2 | Model 3 |
| A | Ref | Ref | Ref | Ref | Ref | Ref | Ref | Ref |
| B | 0.640 (0.354-1.156) | 0.842 (0.454-1.561) | 0.695 (0.382-1.263) | 0.878 (0.472-1.634) | 0.727 (0.423-1.249) | 0.959 (0.545-1.690) | 0.752 (0.436-1.299) | 0.985 (0.557-1.740) |
| C | 1.411 (0.739-2.696) | 1.533 (0.793-2.964) | 1.368 (0.710-2.635) | 1.457 (0.748-2.837) | 1.382 (0.741-2.579) | 1.493 (0.791-2.819) | 1.424 (0.758-2.675) | 1.529 (0.805-2.905) |
| D | 0.473 (0.167-1.340) | 0.475 (0.167-1.357) | 0.526 (0.185-1.500) | 0.523 (0.182-1.500) | 0.537 (0.210-1.376) | 0.535 (0.208-1.381) | 0.538 (0.209-1.385) | 0.527 (0.204-1.365) |
| E | 0.849 (0.297-2.429) | 0.865 (0.298-2.511) | 0.976 (0.294-3.238) | 0.930 (0.271-3.194) | 0.772 (0.271-2.199) | 0.765 (0.265-2.211) | 1.056 (0.329-3.388) | 1.003 (0.301-3.343) |
| F | 0.692 (0.092-5.220) | 0.717 (0.093-5.538) | 0.646 (0.085-4.931) | 0.719 (0.093-5.574) | 0.629 (0.083-4.735) | 0.666 (0.087-5.120) | 0.644 (0.085-4.897) | 0.704 (0.091-5.440) |
| *P* value | 0.274 | 0.430 | 0.474 | 0.617 | 0.398 | 0.500 | 0.434 | 0.523 |

Model 1: adjusted for patient character such as age, sex, location and histologic pattern of tumor.

Model 2: adjusted for contents of procedure such as duration of procedure, combination of EUS, nodal size (short axis), number of aspiration per node, and number of obtained core tissue per node.

Model 3: adjusted for both patient character and contents of procedure.

LN = lymph node; EUS = esophageal ultrasound.
