# Supplementary figures and images for: Are there differences among operators in false-negative rates of endosonography with needle aspiration for mediastinal nodal staging of non-small cell lung cancer?
Source: BMC Pulm Med. 2019 Jan 14;19:14. doi: 10.1186/s12890-018-0774-6 (PMC6332520; doi:10.1186/s12890-018-0774-6)

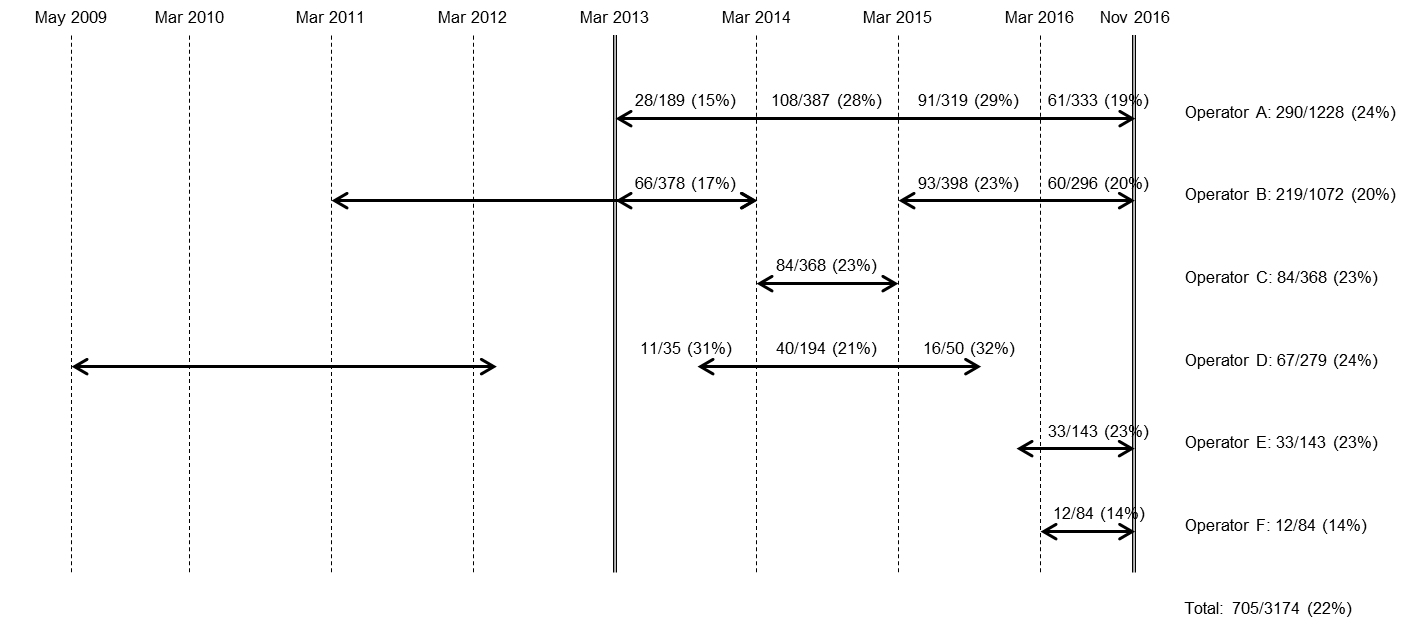

Supplement: Supplementary file 1 — Figure S1. Experience of each operator with EBUS/EUS-NA. Table S1. Baseline characteristics, contents of the procedure, and false negative rate of the total 705 patients analyzed by each operator. Table S2. Baseline characteristics, contents of the procedure, and false negative rate of 681 patients, excluding 24 patients who had false negative result from inaccessible LNs, analyzed by each operator. Table S3. Baseline characteristics, contents of the procedure, and false negative rate of the total 1,737 attempted LNs analyzed by each operator. Table S4. Baseline characteristics, contents of the procedure, and false negative rate of 1,747 attempted LNs (including 10 unattempted accessible LNs) analyzed by each operator. Table S5. Odds ratios for false negative results by operator analyzed by patient with operator A as reference. Table S6. Odds ratios for false negative results by operator analyzed by LN with operator A as reference. (ZIP 91 kb) [file 12890_2018_774_MOESM1_ESM.zip › eFigure_1R1_180606.tif]
